# Critical Role of Spns2, a Sphingosine-1-Phosphate Transporter, in Lung Cancer Cell Survival and Migration

**DOI:** 10.1371/journal.pone.0110119

**Published:** 2014-10-20

**Authors:** Eric Bradley, Somsankar Dasgupta, Xue Jiang, Xiaying Zhao, Gu Zhu, Qian He, Michael Dinkins, Erhard Bieberich, Guanghu Wang

**Affiliations:** 1 Department of Neuroscience and Regenerative Medicine, Medical College of Georgia, Georgia Regents University, Augusta, Georgia, United States of America; 2 Shengjing Hospital, China Medical University, Shenyang, Liaoning, P.R. China; The University of Tennessee Health Science Center, United States of America

## Abstract

The sphingosine-1-phosphate (S1P) transporter Spns2 regulates myocardial precursor migration in zebrafish and lymphocyte trafficking in mice. However, its function in cancer has not been investigated. We show here that ectopic Spns2 expression induced apoptosis and its knockdown enhanced cell migration in non-small cell lung cancer (NSCLC) cells. Metabolically, Spns2 expression increased the extracellular S1P level while its knockdown the intracellular. Pharmacological inhibition of S1P synthesis abolished the augmented cell migration mediated by Spns2 knockdown, indicating that intracellular S1P plays a key role in this process. Cell signaling studies indicated that Spns2 expression impaired GSK-3β and Stat3 mediated pro-survival pathways. Conversely, these pathways were activated by Spns2 knockdown, which explains the increased cell migration since they are also crucial for migration. Alterations of Spns2 were found to affect several enzymes involved in S1P metabolism, including sphingosine kinases, S1P phosphatases, and S1P lyase 1. Genetically, Spns2 mRNA level was found to be reduced in advanced lung cancer (LC) patients as quantified by using a small scale qPCR array. These data show for the first time that Spns2 plays key roles in regulating the cellular functions in NSCLC cells, and that its down-regulation is a potential risk factor for LC.

## Introduction

Lung cancer (LC) is the leading cause of cancer related death in the United States and worldwide [1], [2]. In 2012, there are more than 220,000 new cases and more than 160,000 deaths in the United States alone [1], [3], [4]. LC is a remarkably heterogeneous disease. Its two major forms are non-small cell LC (NSCLC) and small cell LC, among which NSCLC is the most common form which accounts for about 85% of newly diagnosed cases [1], [4].

Genetic abnormalities have linked multiple genes and signaling pathways to NSCLC, including epidermal growth factor receptor (EGFR) family, signal transducer and activator of transcription 3 (Stat3), and phosphoinositide 3-kinase*–*Akt–mTOR pathways [1], [2], [4]. These discoveries have led to uniquely targeted therapies with specific inhibitor drugs such as erlotinib and gefitinib for mutations in EGFR [5] or crizotinib for gene translocation resulting in the EML4-ALK oncogene [6]. Epigenetic variations are also linked to NSCLC [7], [8]. Although its diagnosis and treatment is rapidly evolving, meaningful improvements in outcomes for most NSCLC patients are still elusive [1], [3]. Many patients relapse and form more aggressive metastatic tumors [9]. Thus a deeper understanding of the origins and the molecular mechanisms of metastasis of the disease is urgently needed in order to improve prevention and treatment.

**Figure 1:**
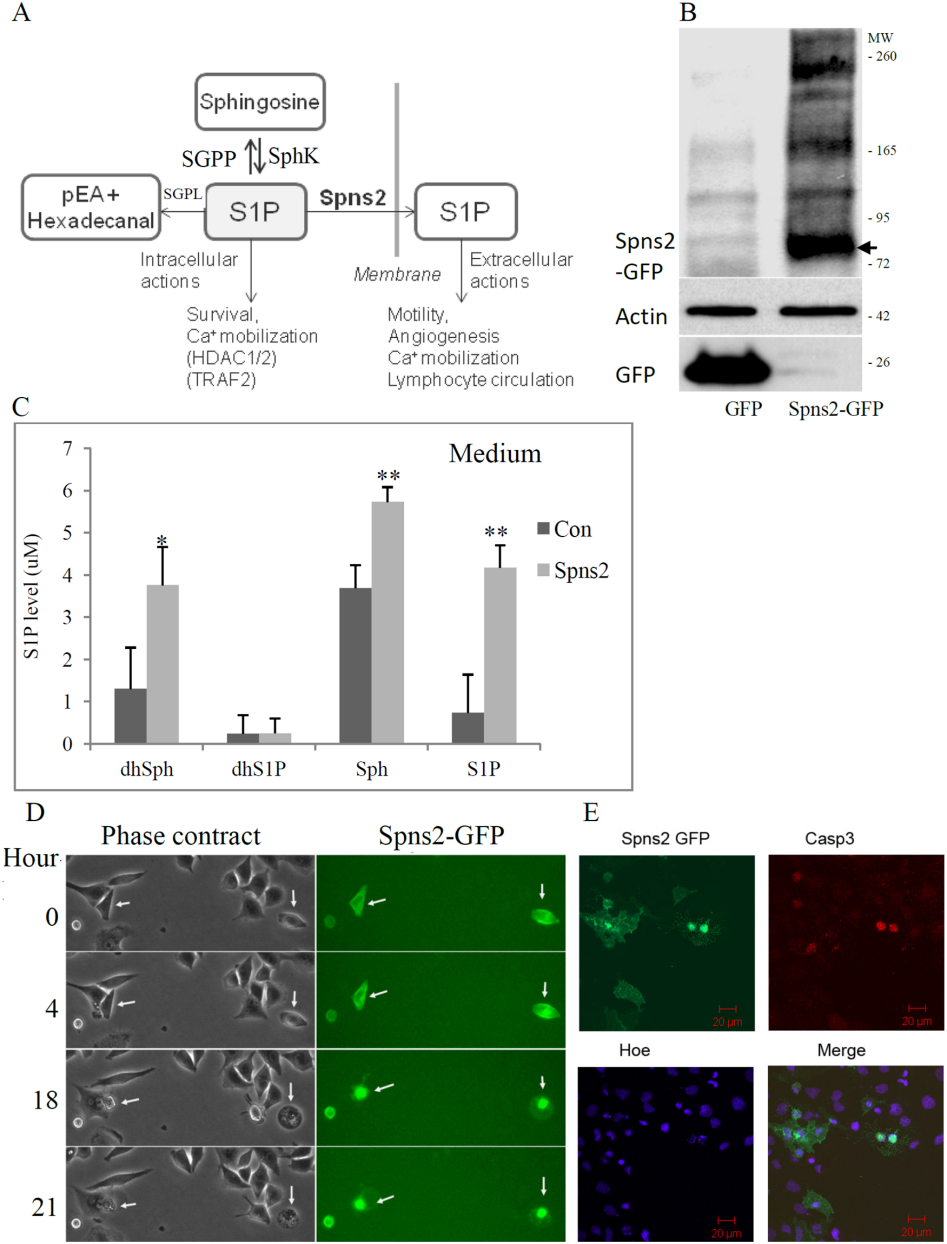
Ectopic Spns2 expression induced apoptosis in A549 cells. (**A**), Schematic representation of the S1P metabolism and function, SGPP, S1P phosphatase; SphK, sphingosine kinase; SGPL, S1P lyase; pEA, phosphoethanolamine. (**B**), Western blot analysis of Spns2-EGFP expression detected by an anti-GFP antibody. β-actin (Actin) was used as a loading control. A549 cells were transfected with Spns2-EGFP and cell lysates collected 48 hours later. The molecular weight of Spns2-GFP is around 84 kD (58+26; arrow). (**C**), Spns2 expression increased extracellular level of S1P, sphingosine (Sph), and dihydrosphingosine (dhSph). Cells were changed into media with delipidated FBS 24 hours after transfection. Another 24 hours later, the media were collected and centrifuged, and supernatant analyzed by lipidomics. (**D**), Time lapse images of Spns2 positive cells. A549 cells were transfected with Spns2-EGFP as in A. 12–16 hours later, cells were placed in an environmental chamber which maintains 37°C and 5% CO_2_ and time lapse images taken at time points indicated. Scale bar is 10 µm. (**E**), Confocal laser scan images of cells immuno-stained with active (cleaved) caspase 3 (Casp3). A549 cells were transiently transfected, fixed and stained with an antibody against cleaved Casp3. Scale bar is 20 µm.

Sphingosine 1-phosphate (S1P) is a potent bioactive signaling molecule that plays vital roles in diverse physiological and pathological processes such as immunity and cancer [10], [11], [12], [13]. It promotes cancer by regulating cell proliferation/survival, migration, angiogenesis, and lymphangiogenesis [10], [14], [15]. One of the enzymes that generates S1P, sphingosine kinase 1 (SphK1) (Fig. 1A), is regarded as an oncogenic enzyme, whose activity can be stimulated by a wide range of agonists, e.g. hormones and growth factors [16], [17]. Conversely, the enzyme that degrades S1P, S1P lyase 1(SGPL1) (Fig. 1A), is down-regulated in prostate cancer [17]. Silencing of SGPL1 enhances cell survival; and enforced SGPL1 expression sensitizes cell to irradiation or chemotherapy [17]. In addition, many aspects of the S1P signal pathways are closely related to tumorigenesis (Fig. 1A) [10], [18]. Extracellular S1P exerts most of its function through five cell surface G-protein-coupled-receptors S1P1–S1P5 [10]. It stimulates different signal transduction pathways in different cell types, as well as within the same cell, depending on the receptors expressed. For example, S1P1 is coupled exclusively *via* Gi protein to activate Ras, mitogen activated protein kinase (MAPK), PI3K/Akt, and phospholipase C pathways [10], [19]. The intracellular S1P, on the other hand, promotes cancer progression in a receptor-independent manner [11], [12], by either mediating calcium release from endoplasmic reticulum, or by interacting with its intracellular targets, such as HDAC and TNF receptor-associated factor 2 (TRAF2) [20]. More importantly, S1P elevation has been implicated as a risk factor for LC in an epidemiological study [21].

S1P is generated intracellularly by SphKs and its cellular level is maintained by a fine-tuned equilibrium among generation, conversion, degradation, and exportation (Fig. 1A). S1P is exported out of the cells by transporter proteins (Fig. 1A). Several ATP-binding cassette (ABC) family members, such as ABCA1, ABCC1, and ABCG1 have been proposed to transport S1P based on observations that their knockdown or pharmacological inhibition decrease S1P release [22], [23], [24], [25], [26]. However, this notion remains controversial since S1P exportation is not altered when these proteins are exogenously expressed in cells or knocked out in mice [18], [27], [28]. Recently, spinster homolog 2 (Spns2), a member of the major facilitator superfamily of non-ATP-dependent transporters, has been shown to transport S1P both *in vitro* and *in vivo* [27], [29], [30], [31], [32], [33], [34]. More interestingly, although reduced in the plasma, the S1P levels are increased in some tissues including lung in Spns2 deficient mice [33], which is consistent with a previous report showing that Spns2 is expressed most abundantly in the human lung [18].

These previous findings prompted us to hypothesize that Spns2 is involved in LC development. We have performed gain-of-function and loss-of-function experiments in NSCLC cells. We have also detected Spns2’s expression level in LC patient samples.

## Materials and Methods

### Materials

Antibodies against phospho-GSK-3β (Ser 9), GSK-3β, phospho-Stat3, Stat3, cleaved Caspase 3, and Survivin were from Cell Signaling Technologies (Danvers, MA). Antibodies against AIF and β-actin were from Santa Cruz Biotechnology (Santa Cruz, CA). The antibody against LC3B was from Abcam (Cambridge, MA). The antibody against SphK2 was from Exalpha Biologicals (Dublin, OH). The fluorescent labeling of inhibitors of caspases (FLICA) kit was from Immunochemical Technologies (Bloomington, MN). SphK inhibitor Ski-1 was from Biovision (Milpitas, CA). The pan caspase inhibitor Z-VAD was from Promega (Madison, WI). The PI3K inhibitor LY294002 was from Cayman (Ann Arbor, MI). And the Jak inhibitor JAKi was from Millipore (Billerica, MA). Human LC real time PCR arrays were from Origene (Rockville, MD).

## Methods

### 

#### Plasmid cloning

Human SPNS2 was PCR amplified from a BAC using primers hSpns2forward: AAG CTT ATG TGC CTG GAA TGC GCC TCG, and hSPns2reverse: GGT ACC AA GAC TTT CAC AGA TGC GGG CGG; and was subcloned into pGEM Teasy vector (Promega, Madison, WI). The fragment was digested using enzymes HindIII and KpnI and cloned into pEGFP-N1 vector (Clontech, Mountain View, CA), resulting in the pSPNS2-EGFP construct. For HA tagged Spns2, primers containing the HA tag were designed. The PCR product was cloned into pCDNA3.1 (Life Tech., Carlsbad, CA, USA) similar to pSPNS2-EGFP.

#### Cell culture and treatment

The human NSCLC cell lines A549 and H1299 (ATCC, Manassas, VA) were generous gifts from Dr. Zhonglin Hao, Cancer Center, Georgia Regents University. The primary human brachial epithelial cells (HBEpC) were from ATCC (Manassas, VA). A549 cells and H1299 cells were maintained in Dulbecco’s modified Eagle’s medium (Cellgro, Herndon, VA, USA) containing 10% FBS (Hyclone, Thermo Fisher Scientific, Austin, TX), and Pen/Strep (Life Tech.). HBEpC were maintained according manufacturer’s instructions. Transfection of Spns2 siRNAs was performed by either Lipofectamine 2000 according to the manufacturer’s instructions (Life Tech.) or electroporated using a Nucleofactor (Lonza, Allendale, NJ).

#### Live cell time lapse imaging, immunocytochemistry, and confocal laser microscopy

Live cell imaging was performed following previous published procedures [35], [36], [37], [38]. Briefly, cells were transfected with Spns2-GFP. Sixteen hours later, the culture was put in a live cell imaging chamber and phase contrast micrographs taken every 30–60 minutes with a Nikon Eclipse TE300 (Nikon Instruments, Inc., Melville, NY, USA. For immunocytochemistry and laser confocal microscopy, the fixed cells were immunostained with antibodies listed and images taken using a Zeiss LSM510 confocal laser scanning microscope equipped with a two photon argon laser at 488 nm(Cy2), 543 nm(Cy3), and 633 nm(Cy5, Alexa Fluor 647), respectively. LSM 510 Meta 3.2 software was used for image acquisition. Adobe Photoshop CS4 was used for background reduction and editing. Images obtained with secondary antibody only were used as negative controls representing the background intensity in a particular laser channel. Antigen-specific immunostaining was quantified by counting cells that showed signals twofold or more above background fluorescence.

#### RNA extraction, RT-PCR, and qPCR

RNA was extracted using the TriZol reagent (Invitrogen). First strand cDNA was generated by the iScript cDNA synthesis kit (BioRad, Hercules, CA, USA). Real time qPCR was performed using SYBR green/Rox qPCR master mix on a PTC-200 Gradient Cycler equipped with a chromo 4 continuous fluorescent detector (BioRad). The primers for qPCR were: human hSpns2 forward: TTA CTG GCT CCA GCG TGA, hSpns2 reverse: TGA TCA TGC CCA GGA CAG; hPOLR2A forward: GGGTGGCATCAAATACCCAGA; hPOLR2A reverse: AGACAC AGCGCAAAACTTTCA; hSphK1 forward: GGC TGC TGT CAC CCA TGA A, hSphK1reverse: TCA CTC TCT AGG TCC ACA TCA G; hSphK2 forward: AGC GTG GTA GCC ACT TCA G, hSphK2 reverse: GAG CAG TGT ACC GAT GCC A; hSGPP1 forward: ATC ATC ATC GGG CTT CAT TTA GC, hSGPP1reverse: GTG CTC CAG GTG TCA AGA GT; hSGPP2 forward, TCA C CG CAC TCC TCA TCG T, hSGPP2 reverse: CCG GGT TGG GCT GTA GTA ATC; hSGPL1 forward: CCT AGC ACA GAC CTT CTG ATG T, hSGPL1 reverse: ACT CCA TGC AAT TAG CTG CCA; hABCA1 forward: TTA AAC GCC CTC ACC AAA GAC, hABCA1reverse: AAA AGC CGC CAT ACC TAA ACT; hABCC1 forward: TTA CTC ATT CAG CTC GTC TTG TC, hABCC1reverse: CAG GGA TTA GGG TCG TGG AT; hABCG1 forward: ACT GCA GCA TCG TGT ACT GGA, hABCG1reverse: CGT CTC GTC GAT GTC ACA GTG; hABCG2 forward: TGA GCC TAC AAC TGG CTT AGA, hABCG2 reverse: CCC TGC TTA GAC ATC CTT TTC AG.

#### Cell viability and cell death assays

Proliferation and apoptosis assays were performed with A549 and H1299 cells transfected with hSPNS2-EGFP or HA-Spns2 as previously described [39], [40], [41]. The number of live cells was measured by a microplate reader using the Cell Counting Kit-8 (CCK8) (Dojindo Molecular Technologies, Inc, which is based on the dehydrogenase activity in viable cells). Briefly, the CCK8 solution was added to the treated cell culture, incubated for 2–4 hours, and their absorption at wavelength 450 nm read by a microplate reader. The absorption at 450 nm was correlated to the live cell number present in the well. The FLICA assay was performed with Spns2-EGFP plasmid-transfected A549 cells using sulforhodamine-labeled fluoromethyl ketone peptide inhibitor (red) according to the manufacturer’s instructions. Briefly, 48 hours post-transfection with the Spns2-EGFP plasmids, the FLICA reagent was added to the medium and the cells incubated for 1 hour at 37°C under 5% CO_2_. The cells were washed once with washing solution and then fixed with 4% *p*-formaldehyde in phosphate buffered saline for further analysis by microscopy.

#### Flow cytometry analysis

Spns2-GFP transfected A549 cells were collected, fixed by 4% paraformaldehyde, and immuno-labeled with Caspase 3 antibody followed by Alexa Fluor 555 secondary antibody. The labeled cells were analyzed by a Becton Dickinson FACSCalibur 4-color analyzers equipped with 4 lasers (BD BioSciences, San Jose, CA).

#### Cell migration assays

Cell migration was measured by wound healing assays as previously published [42]. Briefly, cells were transfected with Spns2 siRNAs or scrambled control by lipofectamine 2000. Cells were grown for 72 hours by which time they had become confluent. A gap of about 400 µm was generated by a pipette tip. The width of the gap was measured at different time points. For Ski-I treatment, 10 µM Ski-I was supplemented 24 hours after siRNA transfection. Cells were cultured for another 48 hours and then assay performed.

#### Sphingolipid and S1P measurement

Cells transfected with Spns2 or Spns2 siRNA were grown for 48 hours and then both cell pellets and media were collected. Cell pellets were washed three times with cold PBS. Culture media were centrifuged for 10 minutes at 4°C to remove floating cells and cell debris. S1P levels in cells and culture media were measured at the Lipidomics Core of the Medical University of South Carolina (under the supervision of Dr. Jacek Bielawski) according to published protocols on a ThermoFisher TSQ Quantum triple quadrupole mass spectrometer, operating in a Multiple Reaction Monitoring (MRM) positive ionization mode, using modified version [43]. Briefly, cell pellets or media corresponding to about 2–3×10^6^ cells, were fortified with the internal standards (ISs: C_17_ base D-erythro-sphingosine (17CSph), C_17_ sphingosine-1-phosphate (17CSph-1P), N-palmitoyl-D-erythro-C_13_ sphingosine (13C16-Cer) and heptadecanoyl-D-erythro-sphingosine (C17-Cer)), and extracted with ethyl acetate/iso-propanol/water (60/30/10 v/v) solvent system. After evaporation and reconstitution in 100 µl of methanol samples were injected on the HP1100/TSQ Quantum LC/MS system and gradient eluted from the BDS Hypersil C8, 150×3.2 mm, 3 µm particle size column, with 1.0 mM methanolic ammonium formate/2 mM aqueous ammonium formate mobile phase system. Peaks corresponding to the target analytes and internal standards were collected and processed using the Xcalibur software system. Quantitative analysis was based on the calibration curves generated by spiking an artificial matrix with the known amounts of the target analyte synthetic standards and an equal amount of the internal standards (ISs). The target analyte/IS peak areas ratios were plotted against analyte concentration. The target analyte/IS peak area ratios from the samples were similarly normalized to their respective ISs and compared to the calibration curves, using a linear regression model. Other sphingolipids, such as ceramide and sphingosine, were also assayed.

#### Statistics

Means and standard deviations were calculated by Microsoft Excel. The statistical significance was calculated using one way ANOVA and Tukey’s post hoc test or repeated measure ANOVA with GraphPad Prism. P<0.05 is considered significant.

## Results

### Ectopic Spns2 expression induces cell death in NSCLC cells

To study Spns2’s function in LC, we used NSCLC cells. We first attempted to establish a Spns2-EGFP stable-expressing A549 cell line, but no such line was obtained (data not shown). Thus we focused on transient expression of Spns2. Western blot analysis using an anti-GFP antibody indicated that Spns2 was expressed as a ∼84 kD fusion protein (58 kD+26 kD; arrow, Fig. 1B). To ensure that the ectopic expressed Spns2 was functional, we measured whether it transported S1P. Lipidomics data showed that the S1P level in the culture medium was increased by 5.6±0.55 fold (Fig. 1C), consistent with previous reports [29], [31], [32], [33], [34] and confirming that the transfected Spns2 was fully functional. Interestingly, extracellular levels of sphingosine (Sph) and dihydrosphingosine (dhsph) were also increased by 1.5 and 2.8 fold, respectively (Fig. 1C), implying that Spns2 might transport these two sphingolipids as well. Different species of ceramide were assayed, but none showed a significant difference when compared to control (Fig. S1A).

**Figure 2:**
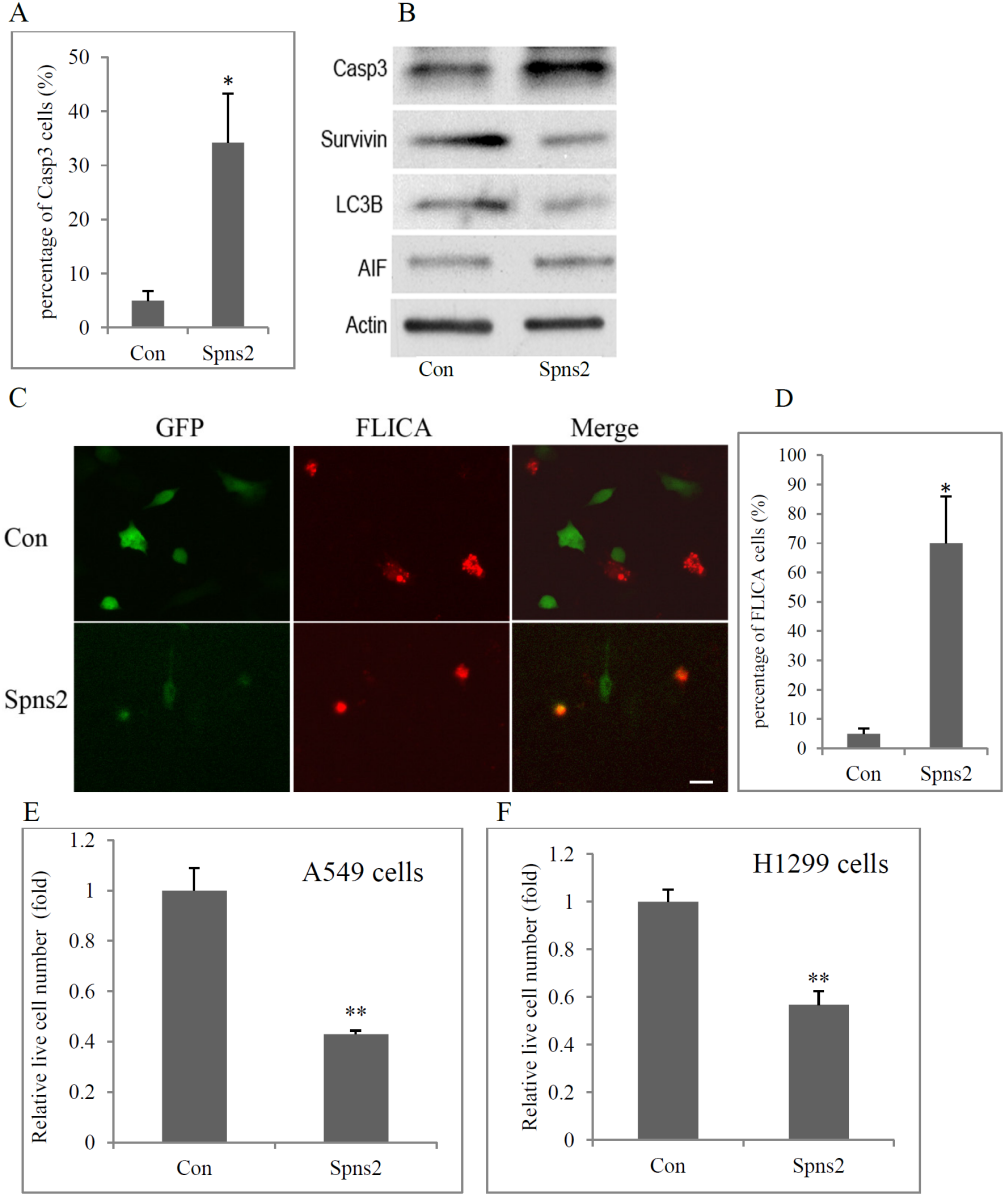
Ectopic Spns2 expression induces apoptosis in NSCLC cells. (**A**), Quantification of Casp3 positive cells shown in D. Vehicle (pEGFP) transfected cells were used as control (Con). N>100 cells are counted from three separate experiments. (**B**), Western blot analyses for cell death markers. A549 cells were transiently transfected as in A. Cell lysates were collected and blotted with the antibodies indicated. Vehicle (pEGFP) transfected cells were used as a negative control and Actin was used as a loading control. (**C**), Representative images of FLICA staining of Spns2 positive cells. Live A549 cells were incubated with FLICA for 1 hour 48 hours after transfection. Cells were fixed and micrographic images taken. Scale bar is 10 µm. (**D**), Quantification of FLICA positive cells shown in C. N>100 cells from three separate experiments were counted double blindly. (**E**), Spns2 expression reduced viable cell number in A549 cells. Cells were transfected as in A. 48 hours later, the relative live cell number was measured by a CCK-8 kit using a microplate reader. N = 4. (**F**), Spns2 expression reduced cell number in H1299 cells. Live H1299 cell number were measured as in I. N = 4. In B, E, H, I, J, error bars represent SD, *, p<0.05, **, p<0.01.

Next we investigated the cellular functions of Spns2 in A549 cells. Live cell imaging showed that Spns2 cells went through dramatic morphological changes and detached from the cell culture dishes (arrows in Fig. 1D), suggesting apoptotic cell death. Thus we immuno-labeled the cells with an antibody detecting activated (cleaved) caspase 3 (Casp3) (Fig. 1E). Double-blinded quantitative analysis indicates that 34.2±9% of the Spns2 positive cells were Casp3 positive (Figs. 1E and 2A). This induction of Casp3 was confirmed by Western blot analysis and flow cytometry; and blocked by a pan caspase inhibitor Z-VAD (Figs. 2B, S1B, and S1C). Induction of Casp3 was reproduced by expression of HA-Spns2 which has a smaller tag (not shown). In line with Casp 3 activation, the anti-apoptosis gene Survivin was significantly reduced (Fig. 2B) [36], [44]. Apoptosis Inducing factor (AIF) and cleaved LC3B were also measured, but none were increased by Spns2 expression (Fig. 2B), suggesting that Spns2 does not induce caspase-independent apoptosis or autophagy.

To further validate that Spns2 induced apoptosis, we performed FLICA (fluorescent labeled inhibitor of pan caspases) assays on living cells. Figure 2C shows typical micrographic images of the assay. Double-blinded quantification demonstrated that 70±15.9% of Spns2 positive cells were FLICA positive (Fig. 2D).

An end result of cell death is reduction in total viable cell number. Unbiased cell counting using a microplate reader (CCK-8) indicated that Spns2 expression resulted in 59±1.2% reduction of viable A549 cells in 48 hours (Fig. 2E). These results were reproduced in another NSCLC cell line H1299 (Fig. 2F).

Taken together, the above data demonstrate that ectopic Spns2 expression induces caspase-dependent apoptosis in NSCLC cells.

### Spns2 expression modulates S1P metabolism

**Figure 3:**
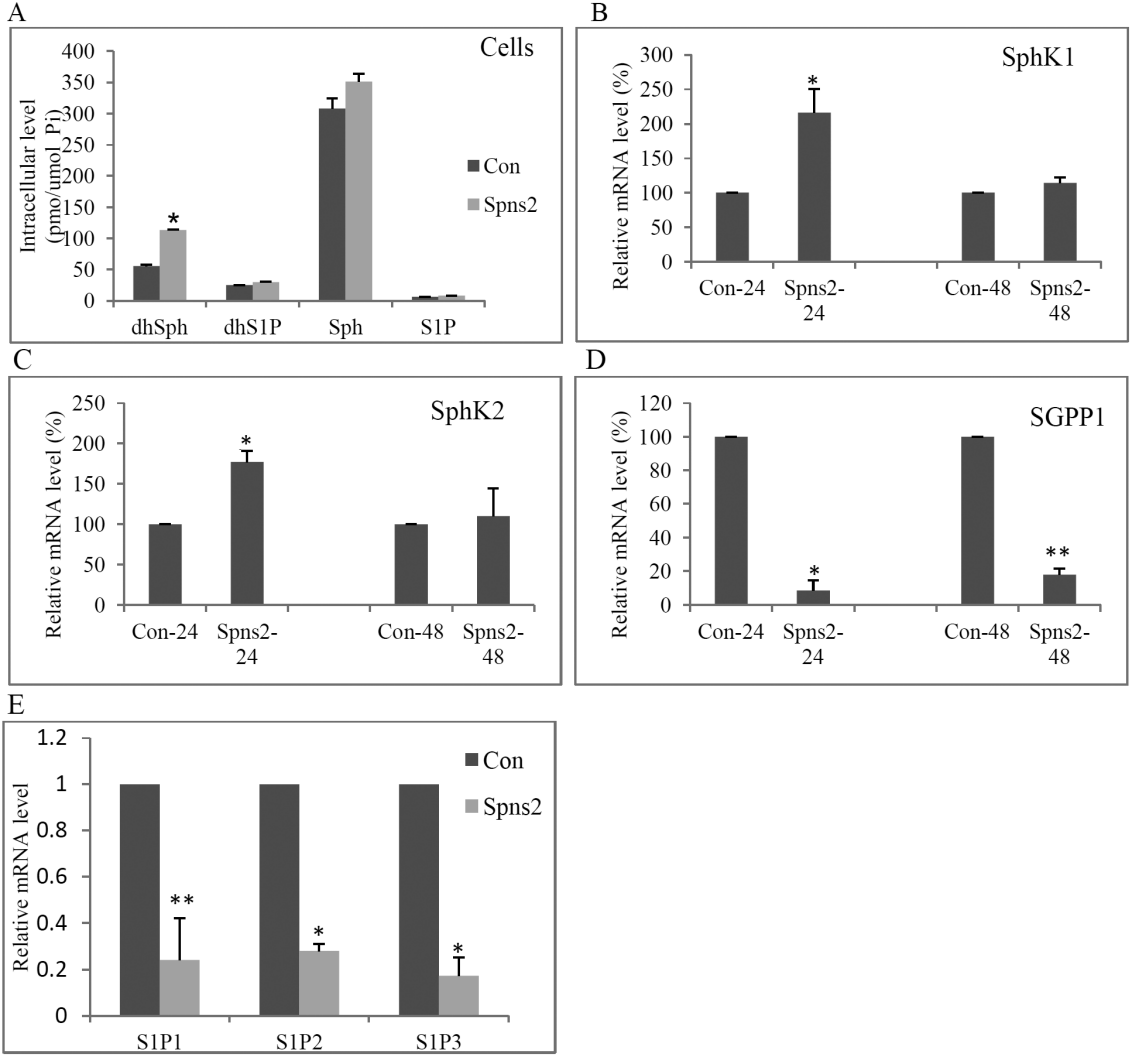
Spns2 regulates S1P metabolism and S1P receptors. (**A**), Spns2 expression did not alter the intracellular level of S1P. A549 Cells were changed into media with delipidated FBS 24 hours after transfection. Another 24 hours later, cells were collected, washed and lipid levels analyzed by lipidomics. (**B**), Spns2 expression led to an acute increase of SphK1 in A549 cells. Cells were transfected, mRNA collected, and real time qRT-PCR performed to measure SphK1. (**C**), Spns2 expression led to an acute increase of SphK2. A549 Cells were treated as in B, except that qRT-PCR performed to measure SphK2. (**D**), Spns2 expression led to reduction of SGPP1 in A549 cells. Cells were treated as in B, except that qRT-PCR performed to measure SGPP1. (**E**), Spns2 expression reduced S1P receptors. Cells were treated as in B, except that qRT-PCR performed to measure S1P1, S1P2, and S1P3. N = 3. Error bars represent SD. *, p<0.05, **, p<0.01. Data were normalized to GAPDH.

We showed that Spns2 expression transported S1P outside of cells (Fig. 1C). Interestingly, the cellular levels of S1P, sphingosine, and different ceramide species were not significantly altered by Spns2 expression, except dhSph and long chain ceramide C20∶1were increased by about 2.0, and 1.4 fold, respectively (Fig. 3A and not shown).

To delineate its underlying metabolic mechanism, we analyzed the key enzymes in sphingolipid metabolism by real time quantitative PCR (qPCR). Both SphK1 and SphK2 mRNA were significantly elevated within 24 hours of Spns2 transfection, and returned to normal levels by 48 hours (Figs. 3B and 3C). Increase of SphK2 expression was confirmed by western blot analysis, except that its protein level remained at a relative high level even at 48 hours after Spns2 transfection (Fig. S1D). This is reasonable since proteins usually take longer time to degrade than mRNA. S1P phosphatase 1 (SGPP1), the enzyme that dephosphorylates S1P, was dramatically reduced to 8.5% of control level within 24 hours, and remained low until 48 hours after transfection (Figs. 3D and S1E). SGPP2 and S1P lyase 1 (SGPL1) were not significantly affected by Spns2 expression (Figs. S1E–S1F). These data indicate that cells alter the expression levels of multiple key enzymes to compensate for S1P exportation mediated by Spns2 expression, including boosting generation (increase in SphKs) as well as halting conversion (reduction in SGPP1).

### Spns2 expression leads to reduction of S1P receptors and multiple pro-survival pathways

As mentioned earlier, S1P is generally a pro-survival factor. To understand why apoptosis was induced when the extracellular S1P was increased after Spns2 transfection, we detected the levels of S1P receptors and found that the mRNA levels of receptors S1P1, S1P2, and S1P3 were all significantly down-regulated after Spns2 expression (Fig. 3E).

**Figure 4:**
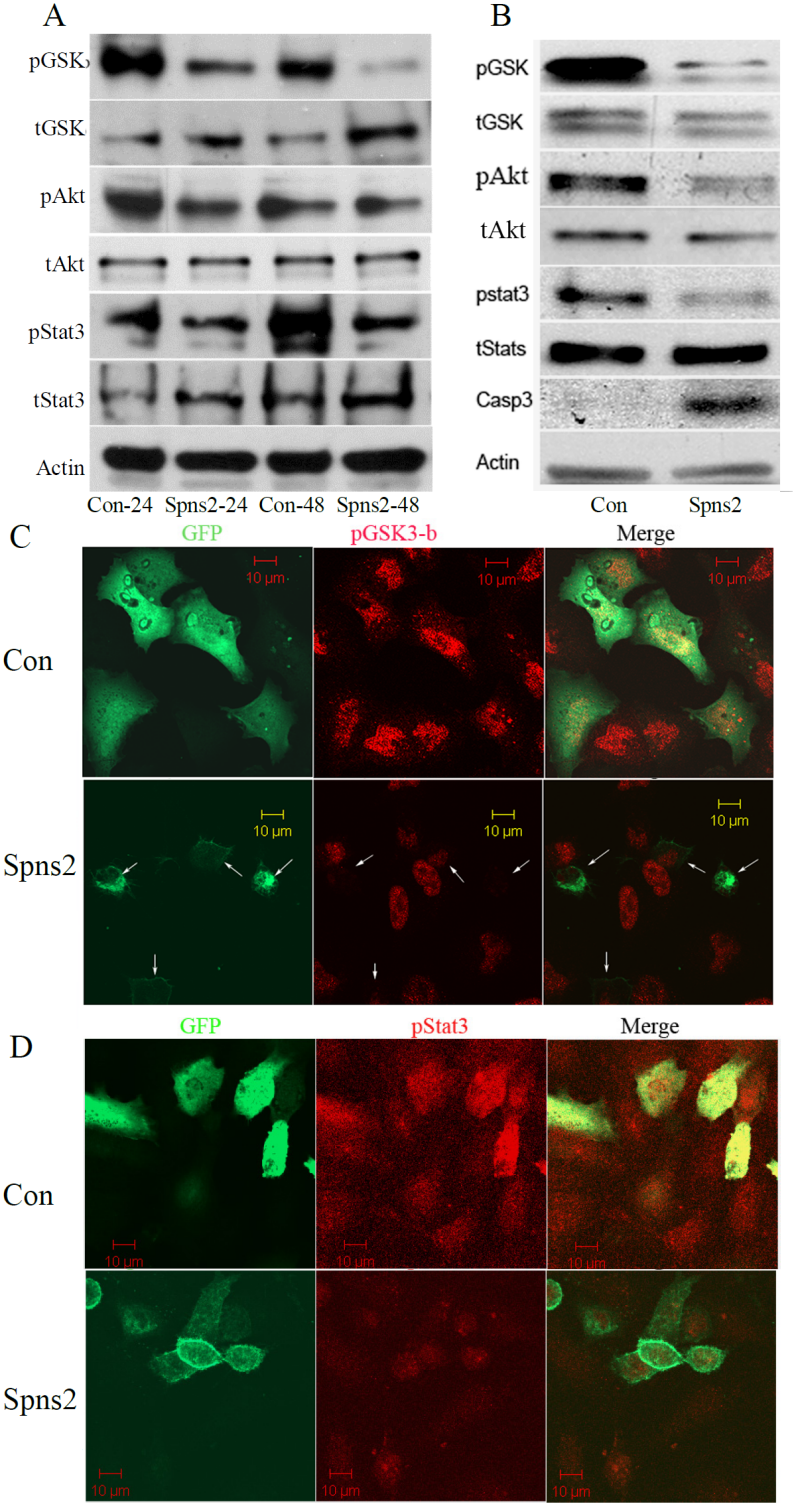
Spns2 expression induces apoptosis by down-regulating pro-survival pathways. (**A**), Western blot analyses on A549 cell samples. A549 Cells were transiently transfected and lysates collected 24 and 48 hours later and blotted with the antibodies indicated. pGSK, phospho-GSK-3β; tGSK, total GSK-3β; pAkt, phospho-Akt; pStat3, phospho-Stat3; tStat3, total Stat3; Actin was used as a loading control. (**B**), Western blot analyses on H1299 cell samples. H1299 cells were transiently transfected as mentioned earlier. Cell lysates were collected 48 hours later and blotted with the antibodies indicated. (**C**), Confocal laser scan images of cells stained for pGSK-3β. A549 cells were transfected with Spns2-GFP and cover slips collected after 48 hours. (**D**), Confocal laser scan images of A549 cells stained for pStat3. Cells were prepared as in C.

To further confirm this observation, we determined the cell signaling pathways downstream of these S1P receptors. Spns2 expression led to drastic reductions of phospho-GSK-3β (pGSK-3β, serine 9) in both A549 and H1299 cells (Figs. 4A and 4B). To validate this observation, we assayed GSK-3β’s upstream activator Akt [35], [45]. Figures 4A and 4B showed that the pAkt levels were reduced by Spns2 expression, indicating that Spns2 impairs the PI3K/Akt pathway. Jak/Stat3 pathway, another signal pathway downstream of S1P receptors, plays crucial roles in cell survival, migration, and immune response [46], [47]. We determined whether Spns2 affects this pathway by measuring Stat3 activity. Western blot analyses showed that the phospho-Stat3 (pStat3) levels were greatly reduced by Spns2 expression in both cell lines (Figs. 4A and 4B). Casp3 was activated by Spns2 in H1299 cells which is consistent with the data obtained from A549 cells (Fig. 4B). Next, we immuno-labeled Spns2-EGFP transfected cells with antibodies against pGSK-3β and pStat3 and found that their fluorescence signals were significantly reduced in all cells in the same culture, whether Spns2 positive or not, when compared to control GFP transfected cells (Con) (Figs. 4C and 4D). This is likely due to extracellular accumulation of sphingolipids, such as Sph. More interestingly, the fluorescent signal for pGSK-3β was further reduced in Spns2-GFP cells than non-transfected cells (arrows in Fig. 4C). These data confirm that these pro-survival pathways were compromised when Spns2 was ectopically expressed.

### Spns2 knockdown increases intracellular S1P level

**Figure 5:**
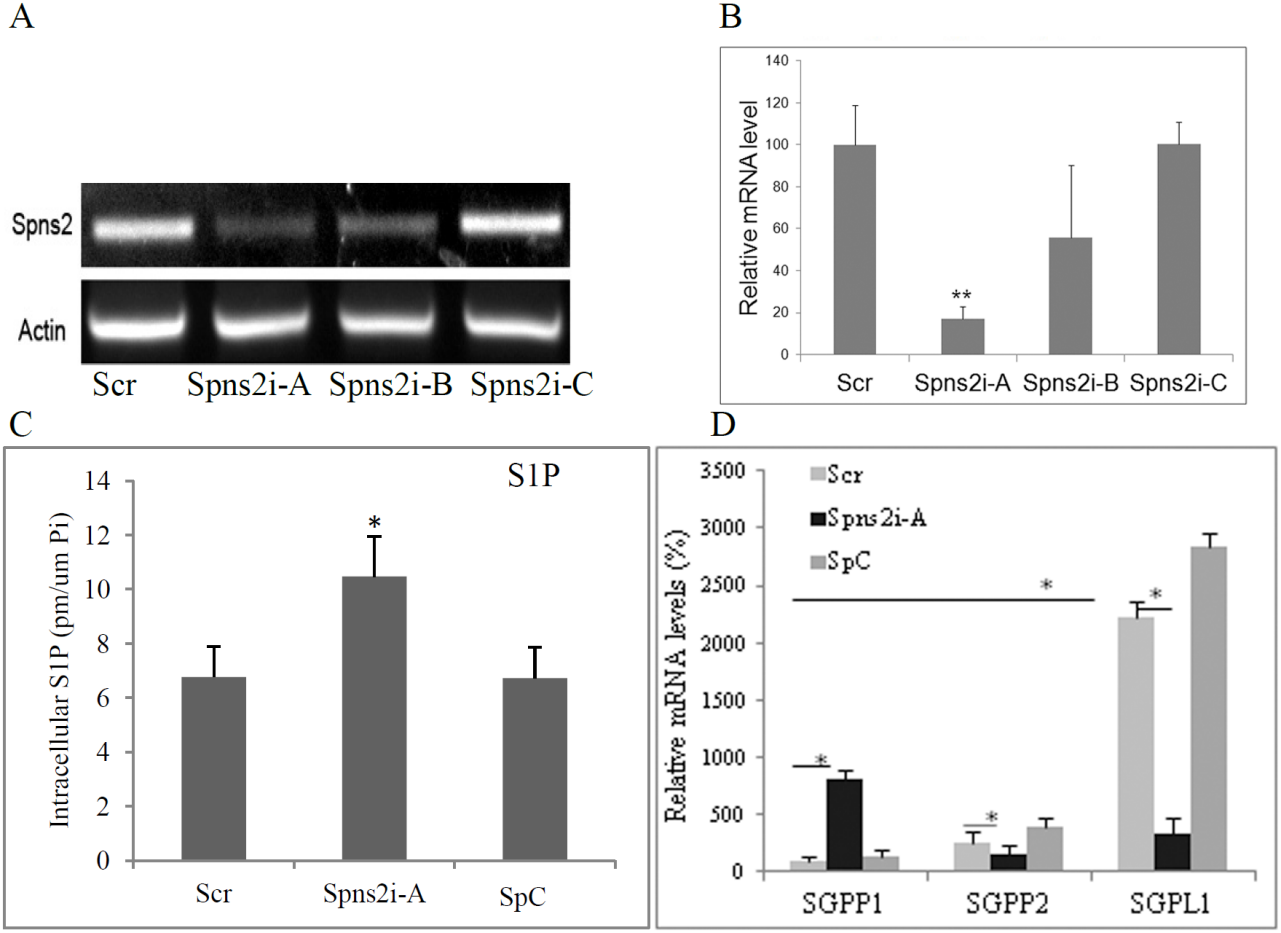
Spns2 knockdown increases intracellular S1P level. (**A**), (**B**), Characterization of Spns2 siRNAs. 20 nM of Spns2 siRNA A, B, and C were transfected individually into A549 cells. 48 hours later total RNA were collected and RT-PCR (A) and qRT-PCR (B) performed to measure Spns2. Scrambled RNA (Scr) was used as a negative control. (**C**), Spns2 knockdown by Spns2i-A significantly increased intracellular S1P level. A549 Cells were transfected with Spns2 siRNA as in A, 24 hours after transfection, cell culture media were changed into media with delipidated FBS. Another 24 hours later, cell pellets were collected and S1P analyzed by lipidomics. N = 3. (**D**), Spns2 knockdown alters the levels of SGPP1, SGPP2, and SGPL1. A549 Cells were treated as in A, except qRT-PCR was performed to measure SGPP1, SGPP2, and SGPL1. N = 4. qPCR data were normalized to GAPDH.

The above data demonstrate that Spns2 regulates S1P metabolism and its ectopic expression leads to apoptosis in NSCLC cells. To further dissect Spns2’s role in LC, we knocked down Spns2 by siRNA. Figures 5A and 5B show that siRNA Spns2i-A significantly reduced Spns2 mRNA level by more than 80%. Thus we used Spns2i-A in our further studies. Scrambled RNA (Scr) and Spns2i-C (hereafter SpC), which did not suppress Spns2 expression, were used as negative controls.

To characterize the effect of Spns2 knockdown on sphingolipid metabolism in NSCLC cells, we determined the levels of sphingolipid and those of their related enzymes. Lipidomics data shows that Spns2 knockdown by Spns2i-A significantly increased the intracellular S1P level, while reduced Sph level, in A549 cells when compared to Scr and SpC (Figs. 5C and S2A). The levels of different ceramide species were not altered by Spns2 knockdown (Fig. S2B). This is consistent with a previous report that S1P deficiency in mice leads to increased level of S1P in the lung [33]. In order to analyze the mechanism of increased cellular S1P, we measured the levels of major enzymes in S1P metabolism. Figure 5D shows that levels of multiple enzymes were altered after Spns2 knockdown by Spns2i-A: SGPP1 was significantly increased by 8 fold; SGPP2 was reduced by ∼50%; and SGPL1 was reduced by more than 80%. Increase in SGPP1 will accelerate S1P conversion to reduce cellular S1P level, which will be counteracted by reduction in SGPP2 and increase in SGPL1. However, SGPL1 was much more abundantly expressed than SGPP1 in A549 cells (22 fold higher) (Fig. 5D). This explains why S1P was increased after Spns2 knockdown. Other enzymes, such as SphKs, were not significantly affected by Spns2 knockdown (Figs. S2C and S2D).

### Spns2 knockdown enhances NSCLC cells migration

Functionally, we first examined Spns2 knockdown’s role on cell survival/proliferation since ectopic Spns2 expression induced apoptosis. Live cell counting assays found that Spns2 knockdown with Spns2i-A did not result in a significant increase in the number of viable cells in either A549 or H1299 cells (not shown), indicating that Spns2 down-regulation does not affect cell survival or proliferation in NSCLC cells.

**Figure 6:**
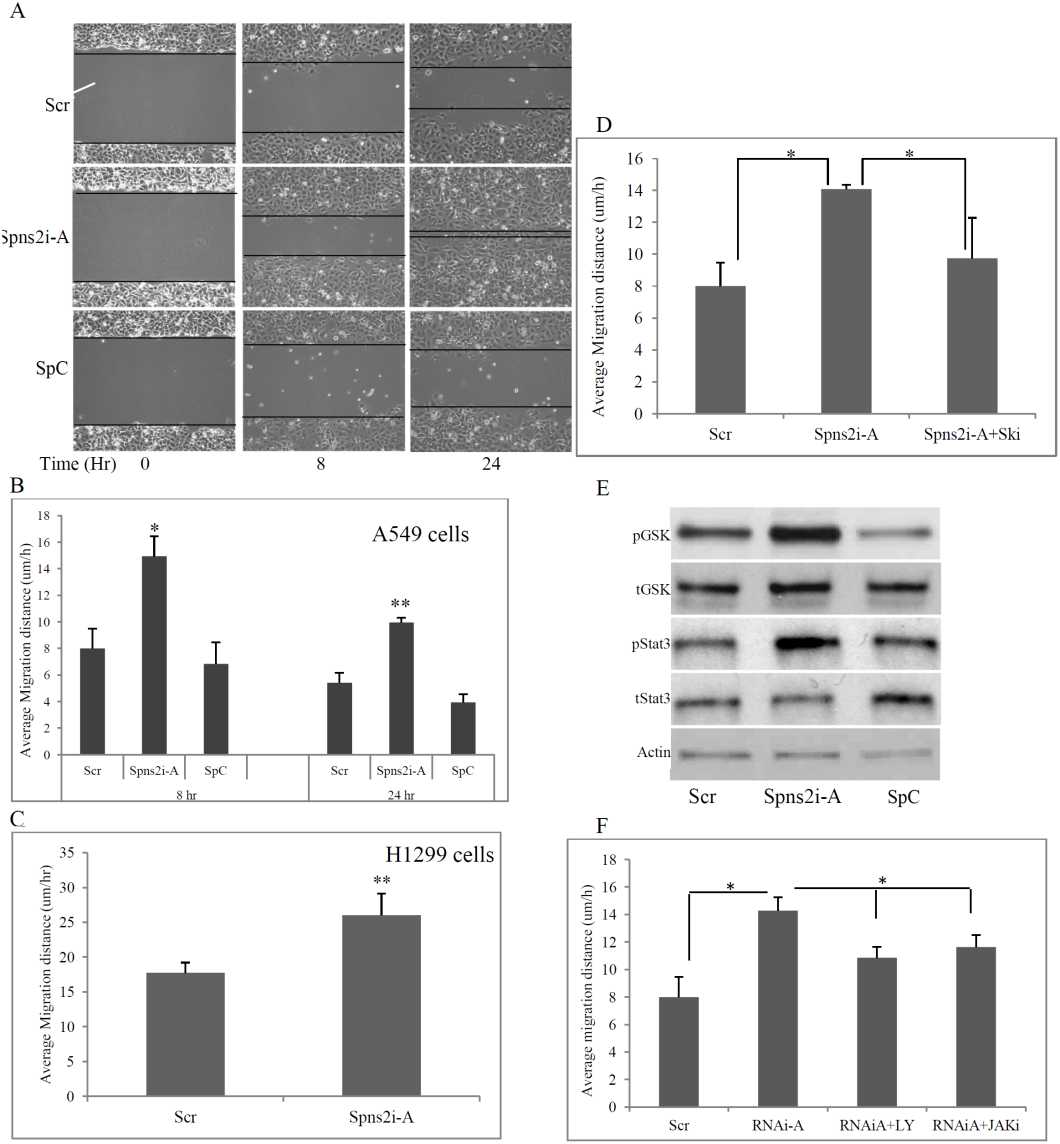
Spns2 knockdown enhances NSCLC cells migration. (**A**), Spns2 knockdown enhanced A549 cell migration. Representative images of the wound healing scratch assays. A549 cells were transfected with Spns2i-A. Scrambled RNA and Spc were used as negative controls. 48 hours later, confluent cells were treated with mitomycin C, and a ∼400 µm gap generated with a pipette tip. Micrographic images were taken at time points indicated. (**B**), Quantification of A549 cell migration after Spns2 knockdown. Average migration speed was calculated using formula v = (G_0_–G_t_)/2t, in which v is velocity or speed; G_0_ is gap size at time 0, G_t_ is gap size at time t and t is time elapsed. N = 6. (**C**), Spns2 knockdown enhanced H1299 cell migration. H1299 cells were treated as in A. Quantification was done as in B. N = 6. (**D**), SphK inhibition by Ski-I eradicated the enhanced migration of A549 cells mediated by Spns2 knockdown. A549 cells were treated as in A, except some cells were treated with the SphK1 inhibitor Ski-I. Cell migration was measured as in B. N = 6. *, p<0.05. (**E**), Spns2 knockdown increased pGSK-3β and pStat3 levels. A549 cells were treated as earlier. Cells were collected and western blot analysis performed to detect for the proteins indicated. (**F**), Inhibition of PI3K/Akt pathway by LY 290004 (LY) and Jak/Stat3 pathway by JAKi partially ameliorated Spns2 knockdown-mediated migration in A549 cells. Cells were treated and migration quantified as mentioned earlier. N = 4.

We then examined the role of Spns2 knockdown on cell migration since intracellular S1P is essential for lung cell migration and motility [48]. Wound healing scratch assays were performed with both A549 cells and H1299 cells. Four hundred µm gaps were generated in confluent cultures and micrographic images taken at 8 and 24 hours post-scratching. Figure 6A shows typical images of the assay in A549 cells. Double blinded quantitative analyses showed that control A549 cells (Scr) migrated at a speed of 8.0±1.5 µm/h and 5.4±0.8 µm/h for the 8 and 24 hour period, respectively (SpC cells have a similar result) (Fig. 6B). However, Spns2 knockdown cells migrated at a speed of 14.9±3.1 µm/h and 10.0±0.3 µm/h in the same time period, representing a 1.86 and 1.85 fold increase, respectively (Fig. 6B). This observation was replicated in H1299 cells (Fig. 6C). To characterize whether this phenomena is specific for LC cells, we transfected human primary brachial epithelial cells (HBEpC) with Spns2i-A and found that Spns2 knockdown did not alter their migration (not shown).

To determine whether enhanced migration is caused by increased cellular S1P, we inhibited S1P generation by a SphK inhibitor, Ski-1. Figure 6D showed that Ski-I treatment eradicated the enhanced migration mediated by Spns2 knockdown, suggesting that cellular S1P is the major reason for augmented migration mediated by Spns2 knockdown in NSCLC cells.

To determine the cell signaling of Spns2 knockdown-induced migration, we measured the Akt/PI3K and Jak/Stat3 pathways. Spns2 knockdown by Spns2i-A significantly increased levels of pGSK-3β and pStat3 when compared to the controls (Fig. 6E), indicating that PI3K/Akt and Jak/Stat3 pathways were activated by Spns2 knockdown. To validate involvement of the two pathways, we supplemented LY294002 (an inhibitor of PI3K) and JAKi (an inhibitor of Jak) into the media. Figure 6F shows that both inhibitors partially reversed the migration mediated by Spns2 knockdown, confirming that PI3K/Akt and Jak/Stat3 pathways play major roles in migration mediated by Spns2 knockdown. These results are consistent with previous reports that both the PI3K/Akt and JAK/Stat3 pathways are crucial for cell migration and motility [42], [47], [49].

### Dysregulation of Spns2 mRNA in LC patient samples

**Figure 7:**
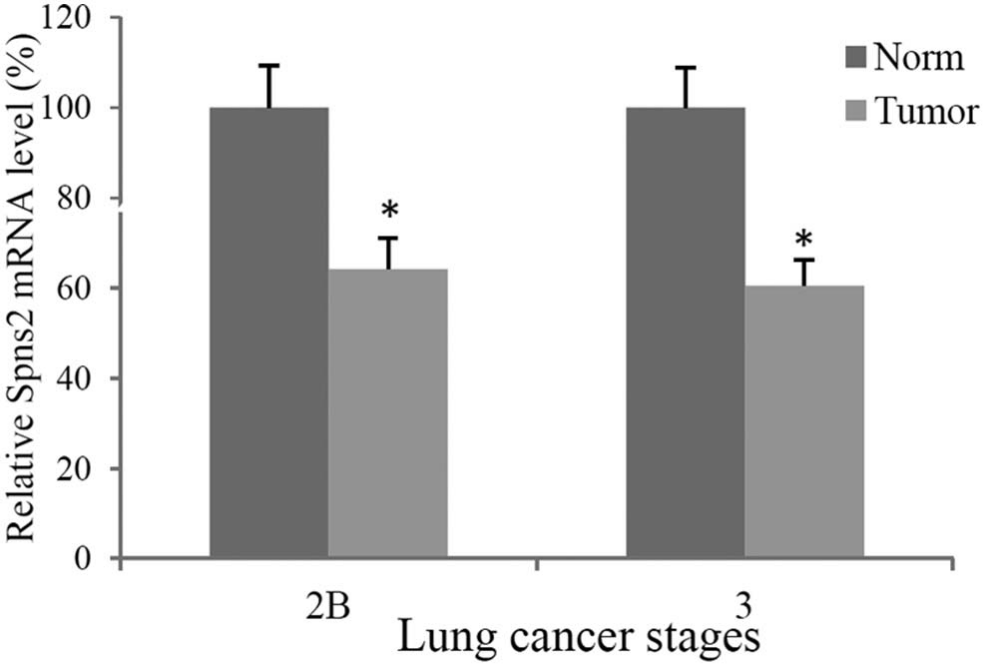
Spns2 is dysregulated in LC patient samples. Real time quantitative PCR of Spns2 in LC samples. Norm, normal adjacent tissues from the same patients; 2B, stage 2B; 3, stage 3. Spns2 levels were normalized to POLR2A levels. Sample sizes are: stage 2B: N = 8; stage 3: N = 6. Error bars represent SD. *, p<0.05.

To build a genetic link between Spns2 and human LC, we determined whether Spns2 expression level is deregulated in patient samples. Real time quantitative PCR using a small scale qPCR array showed that Spns2 mRNA levels were significantly reduced in stage 2B and stage 3 samples when compared to adjacent normal control tissues from the same patients (Fig. 7). Other stages did not show statistical significance, probably due to small sample sizes. There was no stage 4 patient sample in this particular array. This data suggests that Spns2 may be reduced at the mRNA level in advanced LC.

## Discussion

Spns2 was first discovered in zebrafish, in which spns2 mutation led to a migration defect of myocardial precursors and as a result, malformation of the heart [31], [50]. Most recently, Spns2 was found to be essential for lymphocyte trafficking in mice [32], [33], [34]. However it remains unclear whether Spns2 plays a role in tumorigenesis and cancer progression. Thus we set out to test Spns2’s function in cancer. LC was chosen as our focus for several reasons: 1) Spns2 is most abundantly expressed in the lung; 2) lung S1P level is increased when Spns2 is knockout in mice; and 3) S1P has been implicated as an early risk factor for LC in an epidemiology study [21], [27], [33].

Interestingly, our functional studies demonstrate that ectopic Spns2 expression led to apoptotic cell death in NSCLC cells (Figs. 1 and 2). Although the Spns2 transfection efficiency was relatively low (∼20%) (Figs. 1D, 1E, 4C and 4D), mRNA levels of S1PP1 and S1P receptors S1P1-3 were significantly reduced after Spns2 transfection (Fig. 3D and 3E), and so were the protein levels of pGSK-3β and pStat3 (Fig. 4A). One possibility for this phenomenon is that enhanced secretion of the sphingolipids (such as Sph) from the overexpressing cells affected the mRNA levels and pGSK-3β and pStat3 protein levels in none-transfected cells. Despite these molecular changes, only Spns2 transfected cells were apoptotic (Figs. 1D and 1E), which is possibly caused by an interplay of both intra- and extra-cellular signals after Spns2 transfection. Furthermore, we have observed cytoskeletal changes after Spns2 transfection which could be an additional cause for apoptosis.

Conversely, Spns2 knockdown by siRNA led to enhanced cell migration (Fig. 6). Inhibition of S1P synthesis by a SphK inhibitor abolished Spns2 knockdown mediated cell migration (Fig. 6D), suggesting that cellular S1P is the major cause for increased cell migration after Spns2 knockdown.

S1P metabolism is regulated by a complex network of enzymes and factors (Fig. 1A). In this network, changes in any of the components will shift the equilibrium, including Spns2. Unsurprisingly, Spns2 expression dramatically increased the extracellular S1P level and its knockdown the intracellular (Figs. 1C and 3A), which agrees with previous reports [27], [29], [33], [34]. Interestingly, Spns2 seemed to transport sphingosine and dihydrosphingosine as well (Fig. 1C). However, cellular S1P level did not change after Spns2 expression (Fig. 3A), suggesting that cells initiate a compensatory mechanism to make up the S1P loss mediated by Spns2 expression. To delineate the metabolic mechanism, we measured the enzymes involved in S1P metabolism. Our data indicated that Spns2 expression led to acute elevation of SphK1 and SphK2, the enzymes that generate S1P, and reduction of SGPP1, which converts S1P (Figs. 3B–3D, S1D). On the other hand, cells whose Spns2 was knocked-down increased S1P conversion by dramatically increasing SGPP1 (Fig. 5D). However, the levels of SGPP2 and the more abundantly expressed SGPL1 were greatly reduced (Fig. 5D). This may be one of the main reasons why intracellular S1P level was increased after Spns2 knockdown.

To understand why increased extracellular S1P (caused by Spns2 expression) did not show a protective effect, we measured the levels of S1P receptors S1P1-S1P3 and found that they were all significantly down-regulated after Spns2 expression (Fig. 3E). Furthermore, Spns2 expression down-regulated, while Spns2 knockdown activated, the Akt/PI3K and Jak/Stat3 pathways (Figs. 4 and 6E). Inhibition of these pathways partially abolished the enhanced migration, validating that both PI3K/Akt and Jak/Stat3 signaling are directly involved in Spns2 knockdown mediated migration (Fig. 6F). This is consistent with previous publications that both of these pathways are vital for cell migration [47], [49].

In line with our *in vitro* studies, Spns2 mRNA was found to be down-regulated in advanced stage LC patient samples (Fig. 7) when compared to normal adjacent controls from the same patients. This data suggest that Spns2 might be a potential risk factor for LC.

Taken together, we have demonstrated that ectopic Spns2 expression leads to apoptosis and its knockdown results in enhanced cell migration in NSCLC cells. Interestingly, a small scale qPCR array analysis shows that Spns2 mRNA level is reduced in advanced stage LC patients. These observations are of potential significance since reducing apoptosis and enhancing migration are two complementary functions utilized by cancer cells to progress to more aggressive forms. The characterization of Spns2’s function in cancer will not only expand our understanding of S1P delivery and function, but may also contribute to designing new therapeutic strategies to prevent and treat LC.

## Supporting Information

Figure S1 (**A**), Intracellular ceramide profile of the A549 cells after Spns2 transfection. Cells were changed into media with delipidated FBS 24 hours after transfection. Another 24 hours later, the cell pellets were collected, washed with cold PBS for 3 times, and analyzed by lipidomics. (**B**), Flow cytometry analysis of Casp3 (FL2) positive cells in Spns2-GFP and control (GFP) cells. Data shown were based on the GFP positive population. (**C**), The pan caspase inhibitor ZVAD abolished Spns2 mediated cell death. (**D**), Ectopic Spns2 expression increased SphK2 protein level as shown by western blot analysis. (**E**), Ectopic Spns2 expression reduced SGPP1 but not SGPP2 expression as shown by RT-PCR. (**F**), Ectopic Spns2 expression did not alter SGPL1 expression as shown by qPCR.
(PDF)

Figure S2 (**A**), Intracellular Sph was reduced in Spns2 knockdown A549 cells. (**B**), Intracellular ceramide was not altered significantly by Spns2 knockdown. (**C**) and (**D**), Spns2 knockdown did not alter significantly the expression of SphK1 and SphK2.
(PDF)
