# Supplementary figures and images for: Critical Role of Spns2, a Sphingosine-1-Phosphate Transporter, in Lung Cancer Cell Survival and Migration
Source: PLoS One. 2014 Oct 20;9(10):e110119. doi: 10.1371/journal.pone.0110119 (PMC4203763; doi:10.1371/journal.pone.0110119)

**Figure S1**

**A**

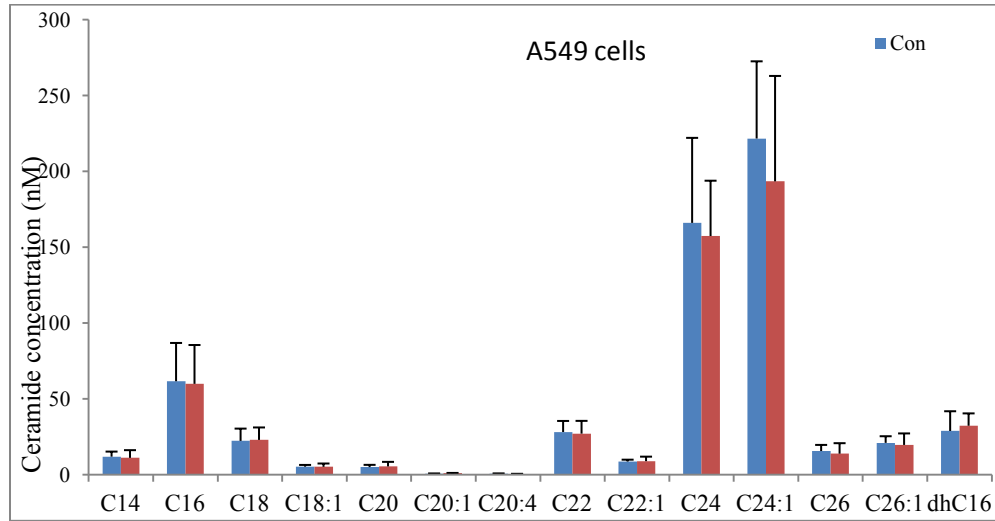

**B**

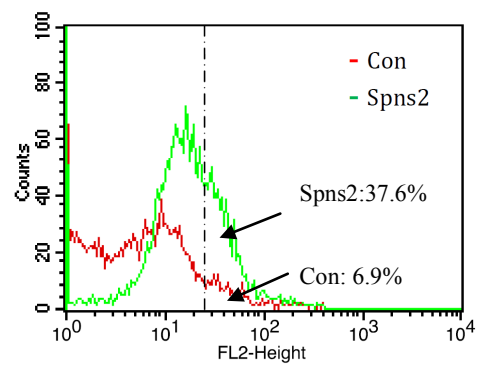

**C**

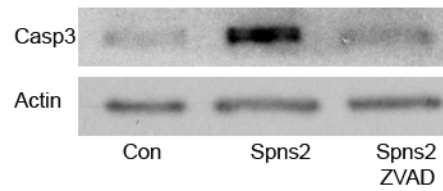

**D**

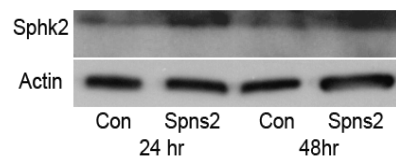

**E**

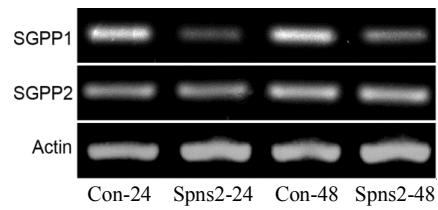

**F**

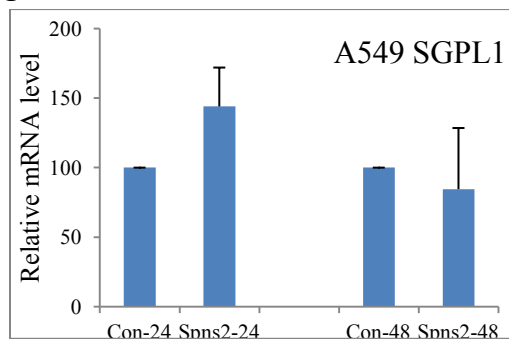

Supplement: Figure S1 — (A), Intracellular ceramide profile of the A549 cells after Spns2 transfection. Cells were changed into media with delipidated FBS 24 hours after transfection. Another 24 hours later, the cell pellets were collected, washed with cold PBS for 3 times, and analyzed by lipidomics. (B), Flow cytometry analysis of Casp3 (FL2) positive cells in Spns2-GFP and control (GFP) cells. Data shown were based on the GFP positive population. (C), The pan caspase inhibitor ZVAD abolished Spns2 mediated cell death. (D), Ectopic Spns2 expression increased SphK2 protein level as shown by western blot analysis. (E), Ectopic Spns2 expression reduced SGPP1 but not SGPP2 expression as shown by RT-PCR. (F), Ectopic Spns2 expression did not alter SGPL1 expression as shown by qPCR. (PDF) [file pone.0110119.s001.pdf]

**Figure S2**

**A**

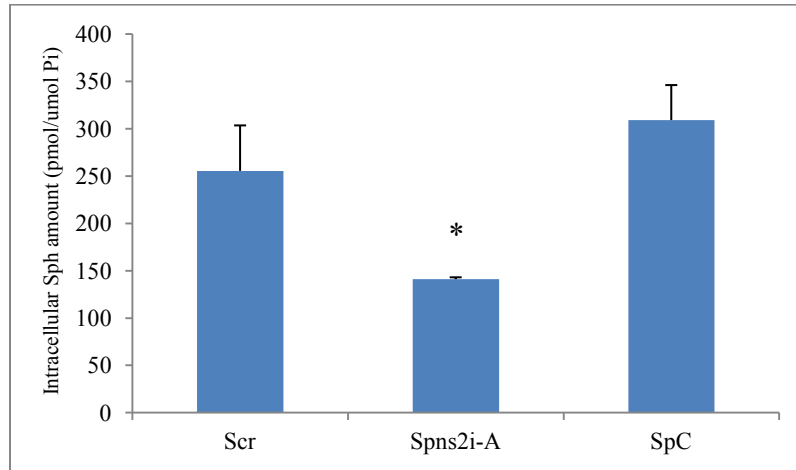

**B**

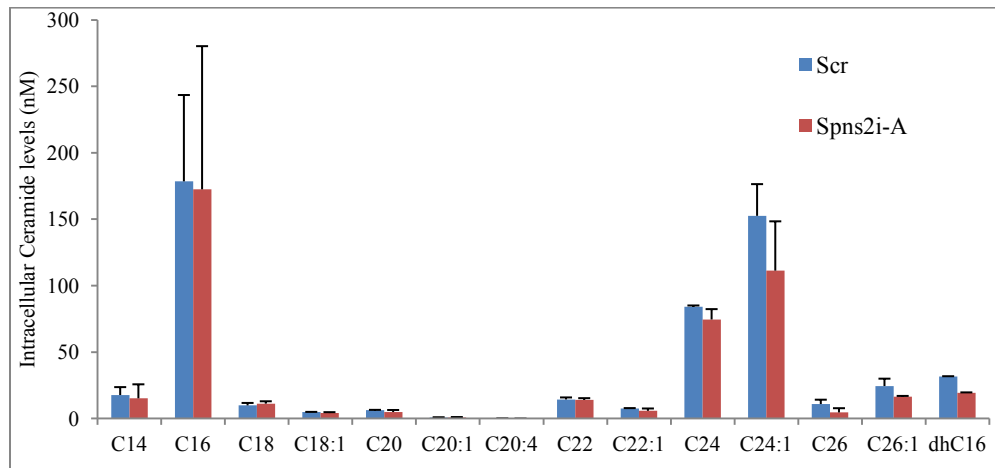

**C**

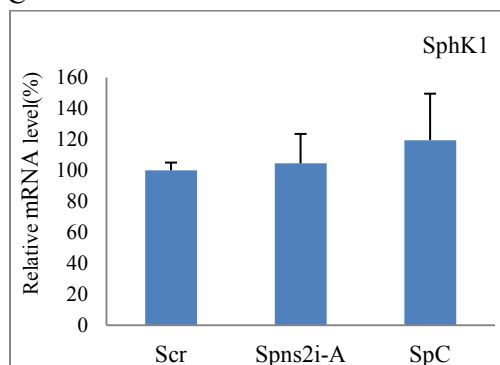

**D**

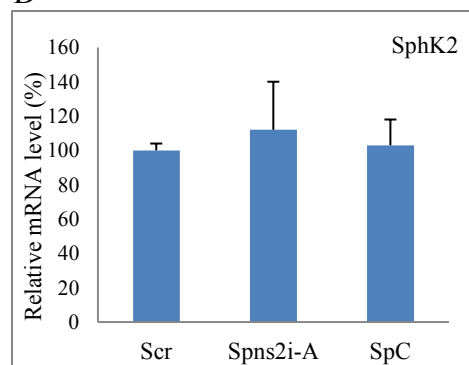

Supplement: Figure S2 — (A), Intracellular Sph was reduced in Spns2 knockdown A549 cells. (B), Intracellular ceramide was not altered significantly by Spns2 knockdown. (C) and (D), Spns2 knockdown did not alter significantly the expression of SphK1 and SphK2. (PDF) [file pone.0110119.s002.pdf]
